# The Genetic Diversity of the Macrophyte *Ceratophyllum demersum* in Backwaters Reflects Differences in the Hydrological Connectivity and Water Flow Rate of Habitats

**DOI:** 10.3390/plants13162220

**Published:** 2024-08-10

**Authors:** Attila I. Engloner, Kitti Németh, Judit Bereczki

**Affiliations:** 1 HUN-REN Centre for Ecological Research, Karolina út 29, H-1113 Budapest, Hungary; 2 National Laboratory for Water Science and Water Security, HUN-REN Centre for Ecological Research, Karolina út 29, H-1113 Budapest, Hungary; 3 Molecular Taxonomy Laboratory, Hungarian National Museum Public Collection Centre, Hungarian Natural History Museum, H-1083 Budapest, Hungary

**Keywords:** gene flow, habitat fragmentation, hydrochory, microsatellite polymorphism, submerged macrophyte

## Abstract

Macrophytes often live in fluvial backwaters that have a variety of hydrological connections to a main river. Since the ability of these plants to adapt to changing environments may depend on the genetic diversity of the populations, it is important to know whether it can be influenced by habitat characteristics. We examined the microsatellite polymorphism of the submerged macrophyte *Ceratophyllum demersum* from various backwaters and showed that the genetic diversity of this plant clearly reflects habitat hydrological differences. The greatest genetic variability was found in a canal system where constant water flow maintained a direct connection between the habitats and the river. In contrast, an isolated backwater on the protected side of the river had the lowest plant genetic diversity. Oxbows permanently connected to the branch system with static or flowing water, and former river branches temporarily connected to the main bed contained populations with moderately high or low genetic variability. The results demonstrate that habitat fragmentation can be a result not only of the loss of direct water contact, but also of the lack of flowing water. Adverse hydrological changes can reduce the genetic diversity of populations and thus the ability of this macrophyte to adapt to changing environments.

## 1. Introduction

In fluvial ecosystems, backwaters can provide various habitats for aquatic organisms. Differences in water flow and connection types to the main river bed and other water bodies result in highly different hydrological conditions and can affect biodiversity in several ways [1,2,3]. Community and population diversity at the taxonomic, functional, and genetic levels within and between habitats may depend on the type of hydrological connections. The latter can range from complete, permanent connection through varied temporal and spatial separation to complete, permanent isolation [2]. Loss of connectivity leads to hydrological fragmentation, greatly reducing freshwater biodiversity and increasing the risk of extinction [4]. The severe consequences of habitat fragmentation on genetic diversity threaten a variety of freshwater organisms around the world [5]. Decreased gene flow and increased vulnerability to genetic drift, inbreeding and selection can both be a consequence of habitat fragmentation [6,7].

Aquatic macrophytes are completely dependent on the water supply of the habitat, which determines the conditions for their life and their colonization [8,9]. Since these plants have both sexual and asexual reproduction, colonization can occur through seeds and vegetative shoot fragments spread by animals, wind and water [10]. Both sexual reproduction and gene flow from distant areas increase the genetic diversity, and thus the potential adaptability of these plants to their environment [11,12]. Although the animals and wind may ensure the dispersal of macrophytes between isolated water bodies, hydrochory (that is, spread by water flow) cannot occur between backwaters without hydrological connections [13,14,15]. Therefore, when hydrochory is exclusive or dominant for a plant species, not only the amount of water in the given habitat, but also the degree of connectivity between different habitats, can be of great importance.

River systems provide a special environment for aquatic plants, enabling long-distance dispersal. Gene flow in linear, unidirectional river channels is easily understood and plant genetic structure in these habitats is well known, as genetic diversity increases downstream, suggesting that flow affects patterns of intrapopulation genetic diversity [16]. However, population genetic diversity in the water bodies of fluvial systems with various hydrological connections and water flow has been little investigated.

*C. demersum* is a cosmopolitan submerged macrophyte known for its low sexual reproduction and dispersal by shoot fragments carried by water flow [17,18,19,20,21,22]. Because microsatellites (i.e., short tandem repeats) are particularly informative and powerful markers for studying the genetic diversity and demographic processes of plants, Engloner et al. [23] developed microsatellite primers for this species and investigated its genetic diversity in lotic and lentic habitats. Compared to the backwaters, the authors showed greater population genetic variability in the main channel and tributaries of the river, where the flowing water provides a permanent longitudinal connection with distant habitats. Although microsatellite polymorphism in the backwaters proved to be diverse, no detailed analysis of the genetic variability of the populations related to hydrological differences has been made for these habitats. Given that aquatic macrophytes mostly live in still or slow-flowing waters [17], a thorough study of plant stands in different fluvial backwaters is essential to understand the effects of habitat hydrology on population genetic diversity, which may affect the ability of macrophytes to adapt to changing environments. The adaptability is particularly important in the backwater systems of rivers, where global climate change can cause significant hydrological changes [24,25].

For the above reasons, the aim of the present study is to explore the microsatellite polymorphism of the populations of the hydrochore *C. demersum* in river backwaters with different hydrological connections to the main bed, and to reveal whether the type of connection can affect the genetic diversity of this species in backwater systems.

## 2. Materials and Methods

### 2.1. Study Sites and Sampling

**Figure 1:**
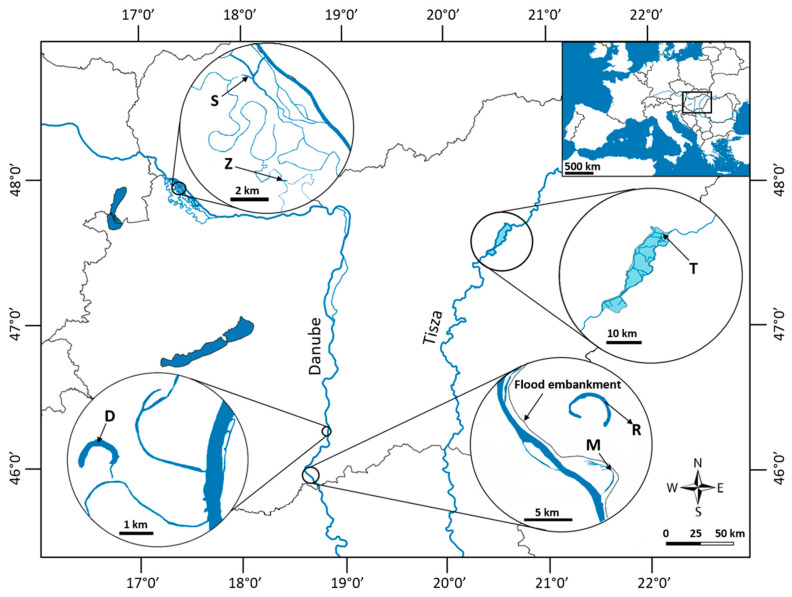
Sampling locations. T—Tisza Reservoir; S—Schisler; Z—Zátonyi; D—Decsi; M—Mocskos and R—Riha backwaters.

Plant material was collected from six aquatic habitats in Hungary, Central Europe (Figure 1). (i) Tisza Reservoir (T), also known as Lake Tisza, is an area of 127 km^2^ flooded by the dammed waters of Tisza River (47°40′12.00″ N, 20°45′30.18″ E). In this reservoir, a network of canals with constant water flow maintains the connection with the river. (ii) Schisler (S) and (iii) Zátonyi (Z) oxbows lie on the Szigetköz water plain, where many branches, islands and backwaters are spread between the main Danube channel and the Moson-arm (47°57′10.00″ N, 17°21′33.90″ E and 47°54′14.80″ N, 17°23′17.00″ E, respectively). Schisler has a direct, close connection with the Danube, which is 1.5 km away. The flow of water is intermittent but frequent. Zátonyi is part of the extensive branch system of the Szigetköz, far from the main river channel, with stagnant water. (iv) Decsi (D) and (v) Mocskos (M) oxbows are former Danube side branches lying on the floodplain of the Gemenc and Béda-Karapancsa Landscape Protection Areas (46°16′53.90″ N, 18°51′48.50″ E and 45°58′12.90″ N, 18°46′23.20″ E, respectively). Both can be temporarily connected to the Danube during the river’s floods, but with different frequencies. In the last fifty years, such an event has occurred only on a third of the days at Mocskos, and only on a sixth at Decsi [26]. (vi) Riha (R) is an isolated backwater on the protected side of the left bank of the Danube near Mohács (river km 1447), supported only by inland inundation (46°0′46.50″ N, 18°45′54.20″ E). The main hydrological differences in the six habitats are briefly summarized in Table 1.

In order to avoid sampling from the same plant (i.e., collecting multiple leaves of the same individual), a distance of at least 30 m was kept between two samples. The amount of plant cover at the habitats T, S, Z, D, M and R allowed for the collection of 20, 16, 8, 13, 10, and 27 plant samples for genetic investigations. Plant material was stored at 4 °C during transport to the laboratory, and then at −80 °C until DNA isolation.

### 2.2. Laboratory Work

DNA was extracted by homogenizing 200 mg frozen plant material in 800 μL CTAB isolation buffer (2%), following the protocol described in [27]. To study the microsatellite polymorphism, ten primer pairs were used according to Engloner et al. [23]. One out of ten loci (namely HME24) was monomorphic in all samples and was therefore omitted from the analyses. During amplification, we used the fluorescent dye-labeled primers described by the authors mentioned above. The amplification procedure from 5 μL of DNA extracts was carried out in 15 μL final reaction volumes containing 10× PCR buffer, 3 mM MgCl_2_, 0.2 mM dNTPs, 0.05 units/μL of Taq DNA polymerase (Taq DNA Polymerase, recombinant, Fermentas), and 0.5 μM of each primer. The cycling conditions were used as provided by Engloner et al. [23]. After amplification, the PCR products were multiplexed in a single reaction and fragment analysis was carried out on an ABI 3130 Genetic Analyser (Hitachi, Tokyo, Japan). Allele sizes were estimated using Peak Scanner software v1.0 (Thermo Fisher Scientific, Waltham, MA, USA).

### 2.3. Statistical Analyses

Micro-Checker 2.2.3 [28] was used for calculating the null allele frequency by Monte Carlo simulation of the expected homozygote frequencies and heterozygote allele size differences. The Micro-Checker analysis did not detect systematic evidence for null alleles at any of the studied microsatellite loci; thus, the whole data set was used for further analyses. The parameters of polymorphism were determined using GenAlEx v. 6.5 [29,30] and Fstat v. 2.9.4 [31]. Among the variability parameters, the number of effective alleles and allelic richness make it possible to compare the genetic variability of populations even with different sample sizes [32,33].

Genetic variation was also characterized by the total allele pool of the backwaters sorted to common (detected in all backwaters) and specific (detected in the given backwater only) alleles.

To reveal the genetic differentiation among plant samples from the six selected habitats, the microsatellite allele frequency data were evaluated by standardized principal component analysis (PCA) using the SYN-TAX 2000 computer program package [34]. This multivariate statistical method is useful for revealing and visualizing differences between objects (in this case plant samples) and groups of objects (samples of the populations) based on all variables (allele frequencies). The distances between objects and object groups displayed as a result of PCA are proportional to the genetic variability between them.

The genetic structure of the populations was analyzed using the Bayesian clustering method [35]. The most probable number of genetically differentiated groups (K) in the populations was estimated and the individuals were assigned to these groups. Structure 2.3.4 was run to carry out these analyses using default settings, with an initial burn in of 100,000 steps and running length of 500,000 steps. In the evaluation of the results, ΔK was computed, which indicates the change in log probability between successive K values [36]. The package ‘pophelper’ in R [37] was applied to compute the ΔK values, average the ten runs of the most probable K value given by Structure, and correct for label switching.

## 3. Results

The level of genetic variability proved to be highly different among the *C. demersum* samples (Table 2). The Tisza Reservoir showed the greatest diversity in terms of almost all parameters (except MG), while the Riha generally had the smallest. The average number of alleles per locus was 2.630; the highest was in the Tisza Reservoir (4.444), and the lowest in the Riha backwater (1.667). The variability parameters corrected for sample size, i.e., the number of effective alleles and the allelic richness, in total were 1.990 and 3.611, respectively. Based on these two parameters and Shannon’s information index, the genetic variability decreases in the following order: Tisza Reservoir > Mocskos, Schisler > Zátonyi > Decsi > Riha. The positions of Mocskos and Schisler are swapped in the row when they are ranked by the number of effective alleles or the allelic richness and Shannon’s information index. A total of 40 different multilocus genotypes were detected: the most in Schisler and the least in Decsi oxbow. Overall, more than 80% of the loci proved to be polymorphic, and this value reached 100% in the Tisza Reservoir and the Mocskos backwater.

**Figure 2:**
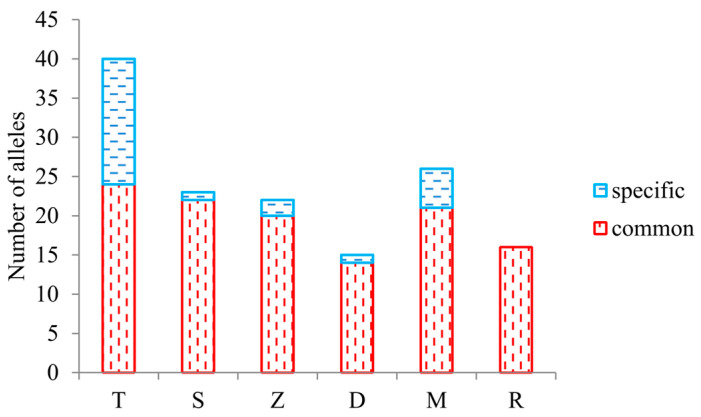
Distribution of alleles among the different classes in the backwaters. Common: detected in all backwaters; specific: detected in the given backwater only. T—Tisza Reservoir; S—Schisler; Z—Zátonyi; D—Decsi; M—Mocskos and R—Riha backwaters.

The allele pool of *C. demersum* amounted to 54 at the nine loci analyzed. The total number of alleles differed largely among the samples (Figure 2). The highest number was observed in Tisza Reservoir (N = 40), the lowest in Decsi backwater (N = 15), and between them it decreased in the following order: Mocskos > Schisler > Zátonyi > Riha. The distribution of alleles between the common and specific classes was also different in the samples. The highest portion of the backwater-specific alleles (N = 16) was observed in Tisza Reservoir, while Riha did not harbor private alleles at all.

**Figure 3:**
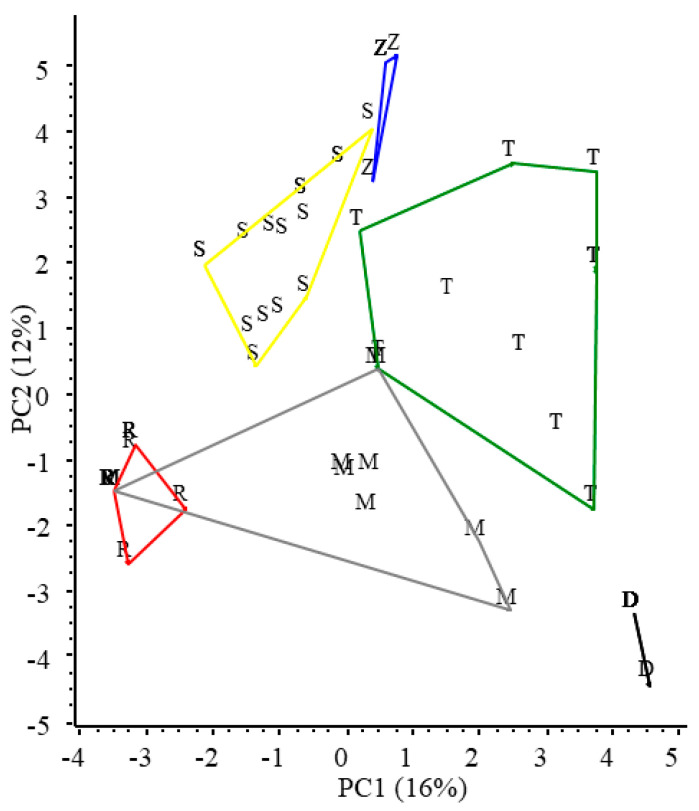
PCA ordination of *C. demersum* samples based on microsatellite allele frequency data. Convex polygons enclose plant samples collected from the same habitats. T—Tisza Reservoir; S—Schisler; Z—Zátonyi; D—Decsi; M—Mocskos and R—Riha backwaters.

The ordination of microsatellite data resulted in well-separated groups of samples taken from the six habitats, and only the samples of Riha and Mocskos backwaters overlapped to some extent (Figure 3). As the areas of the polygons enclosing objects in the scattergram are proportional to the variability between the objects (i.e., the larger the area the higher the variability), the result shows the highest genetic variability of *C. demersum* in Tisza Reservoir, followed by the Mocskos and Schisler oxbows, and the lowest in the other three habitats.

**Figure 4:**
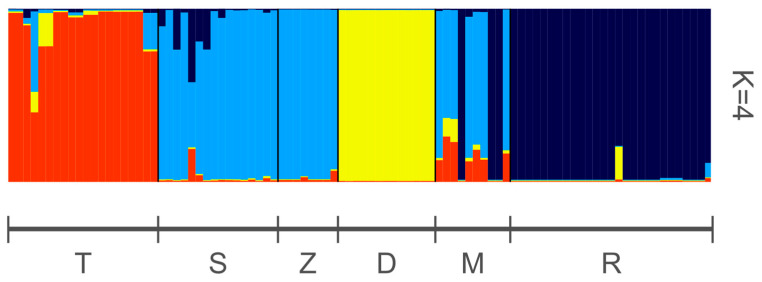
Bayesian assignment of individuals from 6 populations to genetic clusters inferred from the analysis of 9 microsatellite loci for K = 4. T—Tisza Reservoir; S—Schisler; Z—Zátonyi; D—Decsi; M—Mocskos and R—Riha backwaters. The plot represents each individual as a thin vertical bar. The proportion of each color in each column indicates the proportion of an individual’s genome being classified in any of the 4 clusters.

**Figure 5:**
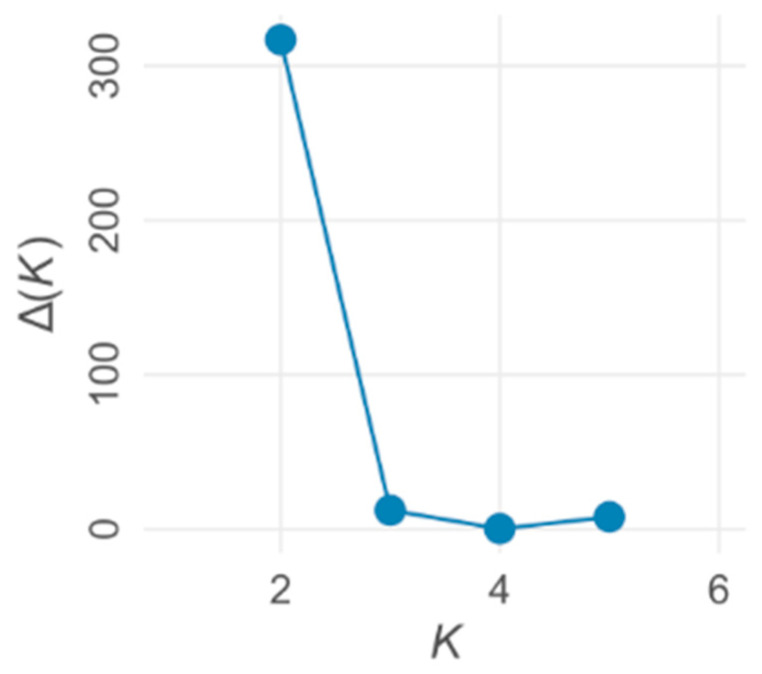
An Evanno method output plot. ΔK obtained by the package ‘pophelper’ in which R indicates the change in log probability between successive K values.

The most probable number of genetically differentiated groups (K) proved to be four in the Structure analysis (Figure 4 and Figure 5). Tisza Reservoir, Decsi and Riha backwaters are clearly separated from each other and from the genetic unit formed by Schisler and Zátonyi, which are not separated by the program at K = 4. Meanwhile, the software defines the Mocskos oxbow as most likely a mixture of the two former backwaters and the Riha.

## 4. Discussion

Since both sexual and asexual reproduction and dispersal by seeds and vegetative shoot fragments can occur in plants, the ratio of these processes, and the predominance of one or the other, highly determines their genetic diversity. In populations of aquatic macrophytes, genetic diversity may be particularly low due to a higher ratio of clonal reproduction and the long-term persistence of the clones [10,38]. Somatic mutations can also increase genetic diversity in clonally spread populations, which is enhanced by rapid clonal growth and a large number of vegetative propagules [38,39,40,41]; however, no somatic mutation associated with *C. demersum* has been reported.

Gene flow from a distant population can also be limited as aquatic habitats are more geographically isolated than terrestrial ones [38]. The wide distribution ranges of many aquatic plants are often considered evidence of high bird-mediated dispersal [10]. Rivers, however, can also provide long-distance dispersal for hydrochore species, resulting in greater genetic variability downstream than upstream [42], and in a main channel and its tributaries than in backwaters [23]. The latter habitats may have various—absent, permanent or temporal—hydrological connections with the main bed and tributaries, providing different opportunities for spreading with water flow. The importance of temporal connection caused by floods in the establishment of coastal populations has already been proven in the marine zones behind the dune systems [14], but the effects of habitat hydrology on the spread of hydrochore macrophytes in river backwaters have not been investigated so far.

This study provides the first results on the genetic diversity of macrophyte populations from backwaters with different hydrological characteristics. Based on the identified multilocus genotypes, these populations were well-separated, and only the Mocskos oxbow appeared to be a home to the genetic mixture of the individual samples of two upstream backwaters (Schisler and Zátonyi) and the closest water body (Riha). Downstream gene flow through the main river channel is easy to understand [42], but the genetic mixing between the Mocskos and Riha backwaters, separated by the embankment, may have two explanations. These two water bodies are very close to each other and were located on the same floodplain before the river regulation. Therefore, the partially shared genetic makeup is either the remnants of the former hydrological connection or a sign of water-independent spread. If the latter occurs at all, it should be to a low degree, given that the distribution of this species relies on vegetative fragments rather than pollen or seeds [20,22].

The variability revealed in the microsatellite polymorphism shows that the genetic diversity of the *C. demersum* populations clearly reflects the habitat hydrological differences. The greatest genetic variability was found in the canal system (Tisza Reservoir), where the constant water flow maintains a direct connection between the habitats and the river. In contrast, macrophytes from the isolated backwater, located on the protected side of the river and supported only by inland water (Riha), showed the lowest genetic diversity. The genetic variability of the other investigated backwater populations fell between this maximum and minimum, depending on the strength of hydrological connectivity and the rate of water flow. In the backwater permanently connected to the branch system, but with static water (Zátonyi), the genetic diversity of the population is moderately low, just as in the former river branch temporarily connected to the main bed during rare flood events (Decsi). Greater genetic variability can be observed in an earlier side branch where flooding is more frequent (Mocskos), and in a backwater with a permanent, close connection to the river, where water can often flow (Schisler).

This clear correlation between habitat hydrology and the genetic variability of a macrophyte with low sexual reproduction and mainly hydrochore spread may mean that adverse hydrological changes may reduce the genetic diversity of these populations. Harmful changes in habitat hydrology can be the result of both global climate change [24,25] and any other human intervention [12]. Habitat fragmentation, in the sense that it impedes gene flow from distant areas [6], can be a result of these plants not only through the loss of direct water contact, but also through the lack of flowing water. In fact, as our results demonstrated, habitats that are periodically totally isolated but frequently flooded by a river may allow for greater macrophyte genetic diversity than those that are connected to distant water bodies by permanent but standing water. As population genetic diversity can greatly affect the ability of plants to adapt to changing environments, it is of great importance to know how this genetic diversity depends on different habitat characteristics.

The correlation between habitat hydrology, genetic variability, and the adaptability of plants to changing environments also draws attention to the importance of conservation implications and the need for possible management strategies. Genetic diversity and adaptive potential (i.e., genetic recovery) can be increased through habitat reconnection or human-assisted translocations [5]. However, the resilience of the reintroduced seeds and plant material may be problematic due to future environmental stress, so the design of mixed propagation lines may become necessary [43]. In fluvial systems, the increase in the length and number of free-flowing river sections can be a solution to restoring habitats and protecting biodiversity [44].

## 5. Conclusions

In fluvial systems, macrophytes face the hydrological variability of their habitat, which describes differences in water flow and connection types to the main river bed and other water bodies. Examination of the microsatellite polymorphism of the hydrochore macrophyte *C. demersum* from different backwaters showed that the genetic diversity of this plant clearly reflects habitat hydrological differences. Population genetic variability is greatest if constant water flow maintains a direct connection between the habitats and the river, while plant genetic diversity is lowest in a completely isolated backwater. Furthermore, oxbows that are periodically totally isolated but frequently flooded by a river may allow for greater macrophyte genetic diversity than those that are connected to distant water bodies by permanent but standing water. Habitat fragmentation can therefore be the result not only of the loss of direct water contact, but also of the lack of flowing water. Since the ability of plants to adapt to a changing environment depends on the genetic diversity of their populations, which is, however, determined by the degree of gene flow from distant areas, the fragmentation of a habitat increases the vulnerability of these populations. Mitigating the effects of adverse hydrological changes resulting from global climate change and any other human activities may require conservation interventions and management strategies. Of course, the ratio of sexual and asexual reproduction and the dispersal of seeds and vegetative shoots are different in macrophytes; therefore, the effects of hydrological connectivity and water flow on population genetic variability should be investigated in additional species.

## Figures and Tables

**Table 1:** A brief summary of the main hydrological differences in the six investigated habitats.

	Connection to the Riverbed	Flow of Water
Tisza-Reservoir (T)	permanent, close	permanent
Schisler (S)	permanent, close	temporary
Zátonyi (Z)	permanent, distant	absent
Decsi (D)	temporary, rare	temporary
Mocskos (M)	temporary, moderately rare	temporary
Riha (R)	absent	absent

**Table 2:** The parameters of variability based on 9 microsatellite loci.

	N	N_a_	N_e_	AR	I	MG	%P
Tisza-Reservoir (T)	19.778	4.444	2.605	3.726	1.023	10	100.00
Schisler (S)	15.778	2.556	2.311	2.529	0.781	13	77.78
Zátonyi (Z)	8.000	2.444	1.873	2.444	0.630	3	77.78
Decsi (D)	13.000	1.667	1.665	1.667	0.462	2	66.67
Mocskos (M)	10.000	2.889	2.125	2.817	0.804	8	100.00
Riha (R)	27.000	1.778	1.359	1.505	0.285	5	66.67
Total	15.593	2.630	1.990	3.611	0.664	40	81.48

N—sample size; N_a_—average number of alleles per locus; N_e_—the number of effective alleles; AR—allelic richness (N = 8); I—Shannon’s information index; MG—the number of multilocus genotypes; P%—percentage of polymorphic loci on the basis of the 95% criterion.

## Data Availability

Data are contained within the article.
